# Phytoremediation: Sustainable Approach for Heavy Metal Pollution

**DOI:** 10.1155/2024/3909400

**Published:** 2024-10-12

**Authors:** Abhijit Kumar, Mishika Dadhwal, Gunjan Mukherjee, Apeksha Srivastava, Saurabh Gupta, Vishal Ahuja

**Affiliations:** ^1^University Institute of Biotechnology, Chandigarh University, Gharuan, Punjab, India; ^2^Department of Biotechnology, Himachal Pradesh University, Shimla 171005, Himachal Pradesh, India; ^3^Department of Microbiology, Mata Gujri College (Affiliated to Punjabi University), Fatehgarh Sahib 140406, Punjab, India; ^4^University Centre for Research & Development, Chandigarh University, Gharuan, Punjab, India

**Keywords:** heavy metals, hyperaccumulator, phytoremediation, pollutants, toxicity

## Abstract

Rapid industrialization, mining, and other anthropogenic activities have poisoned our environment with heavy metals, negatively impacting all forms of life. Heavy metal pollution causes physiological and neurological disorders, as heavy metals are endocrine disrupters, carcinogenic, and teratogenic. Therefore, it becomes mandatory to address the challenge of heavy metal contamination on a global scale. Physical and chemical approaches have been employed for pollutant removal and detoxification, but these methods cannot be adopted universally due to high cost, labor intensiveness, and possible negative impact on natural microflora. Phytoremediation is one of the preferred and safest approaches for environmental management due to its high efficiency and low cost of investment. The plant can uptake the pollutants and heavy metals from water and soil through an intense root network via rhizofiltration and process via phytostabilization, phytovolatilization, and accumulation. At a cellular level, the phytoremediation process relies on natural mechanisms of plant cells, e.g., absorption, transpiration, intracellular storage, and accumulation to counter the detrimental effects of pollutants. It is widely accepted because of its novelty, low cost, and high efficiency; however, the process is comparatively slower. In addition, plants can store pollutants for a long time but again become a challenge at the end of the life cycle. The current review summarizes phytoremediation as a potential cure for heavy metal pollutants, released from natural as well as anthropogenic sources. It will provide insight into the advancement and evolution of advanced techniques like nanoremediation that can improve the rate of phytoremediation, along with making it sustainable, cost-effective, and economically viable.

## 1. Introduction

Industrialization, over the last century, has an exponential growth, thus increasing the consumption of natural resources and in turn leading to a wide variety of environmental issues. Heavy metal contamination is one of the primary effects of various anthropogenic activities (besides natural processes) that pose a major threat to our environment [1]. The major anthropogenic activities responsible for heavy metal pollution include mining, municipal waste, and agricultural and industrial runoff. Over the last few decades, various heavy metals such as arsenic (As), zinc (Zn), copper (Cu), lead (Pb), chromium (Cr), mercury (Hg), nickel (Ni), and cadmium (Cd) have been accumulating in the soil as well as water bodies that pass trophic levels and responsible for toxicity [2]. The contamination of soils with heavy metals can alter the respective ecosystem as the polluted soils become unfit for crop cultivation and will impose toxic effects on other life forms [3]. Based on the activities, some of the sites have become the hotspots of heavy metal contamination, e.g., the soil around coal mines and petrochemical industries that tend to have high concentrations of heavy metals [4]. Statistics show that each year, approximately 5 million tonnes of Pb, 3.4 million tonnes of Cu, 15 million tonnes of Mn, and 1 million tonnes of Ni are released into the soil globally [5] and contaminate the surrounding environment. Hrazdan River, Armenia, is one of the examples of heavy metal pollution in natural resources where contaminants have exceeded the safe levels required [3, 6]. In Bangladesh, agricultural products grown around the industrial areas contain higher concentrations of As, Cd, Cr, and Pb, than permitted [7]. For example, methylmercury chloride is seen to induce a central nervous system (CNS) injury in rats at different concentrations [8]. Minamata disease is another example of mercury poisoning that causes neurological and congenital disorders [5]. Heavy metal pollution in water resources of Uttar Pradesh, India [9], and Damodar River basin, India [10], Lahore, Pakistan [11]; wastewater irrigated vegetables in Bahawalpur, Faisalabad, Gujranwala, Lahore, Multan, Sahiwal, Sargodha, and Rawalpindi (Pakistan) [12]; Nandan Pb–Zn mining area (Guangxi, China) [13]; soil near Carmina mine site (Asturias, NW Spain) [14]; and Aznalcóllar mine (Seville, SW Spain) [15] are some of the prominent cases of heavy metal pollution in the intrusion of pollutant in food and other natural resources.

These heavy metals are highly toxic, nonbiodegradable, and present in the soil in different forms and confirmation *viz* dissolved ions (such as Cd⁺, CrO_4_^2^^−^, Pb^2^⁺, and MoO_4_^2^^−^), exchangeable ions (such as Cu^2+^, Zn^2+^, Cd^2+^, and Ni^2+^), and organic complexes (such as Pb^2+^ and Hg^2+^ that bind to dissolved organic matter in the soil) [16]. It can also form toxic complexes with methyl and ethyl compounds that can accumulate in various trophic levels in a food chain [5]. From water and soil, the heavy metals can intrude at different trophic levels and have toxic effects. According to the 2021 update of the Public Health Impact of Chemicals: knowns and unknowns by WHO, it was estimated that approximately 1 million lives were lost to lead exposure in 2019 [17]. As and Cr exposure are also believed to have carcinogenic effects in both adults and children [18]. By soil and water bodies, these pollutants enter biological systems mainly via crops and seafood and impose lethal effects even in low concentrations. The major issue with heavy metals and associated pollutants is the limited range and sensitivity of the detection system. Several methods have been described for heavy metal detection systems, which are described in Table 1.

Various remediation methods have been discovered to tackle the issue of heavy metal contamination of ecosystems. The techniques used for heavy metals and associated complex decontamination and treatment have been classified into physical methods, chemical methods, and biological methods. Physical approaches rely on sedimentation, filtration, drying, crushing, adsorption, and physical entrapment of metal and pollutants from contaminated soil and water. Chemical approaches employed electroplating, ion exchange, and electrokinetic extraction, and soil flushing methods for pollutant removal. The major challenges with physical and chemical methods are cost, efficiency, and toxic residue generation. In contrast, biological methods have been considered safe and eco-friendly methods that work on the biotransformation, bio-absorption, and degradation of pollutants and heavy metals using microorganisms (bioremediation) and plants (phytoremediation) [16, 30]. Each method has its benefits and limitations, which have a direct influence on its application under various circumstances (Table 2).

Bioremediation approaches employ microorganisms and metabolites (enzymes) as biological agents to transform and degrade or accumulate pollutants including heavy metals in biomass. Microbial growth, enzyme catalytic properties, and some environmental physicochemical factors such as salinity, pH, and temperature modulate the bioavailability and natural transformation. Microorganisms including archaebacteria, protozoa, fungi, and algae have already been reported to interact with heavy metals and alter their forms and availability via biomineralization, bioprecipitation, biomethylation, and bioleaching [31]. *Bacillus subtilis, Escherichia coli, Aspergillus niger, Cladophora glomerata, Saccharomyces cerevisiae,* and *Chlorella vulgaris* are some of the widely researched microorganisms that contributed to the bioremediation of heavy metals [30].

Similarly, phytoremediation is the use of plants as catalysts to remove pollutants from soil and water. Some plants including *Alyssum, Azolla, Berkheya, Corrigiola, and Eleocharis* have the tendency to accumulate higher amounts of heavy metals naturally (also called hyperaccumulators), without showing the signs of any toxicity [32, 33]. Plants also have more or less cellular mechanisms as microorganisms, but the amplitude is much higher and hence can accumulate large amounts of pollutants for longer duration. Phytoremediation has garnered quite some attention over the past, thus leading to the discovery of many plants that can be exploited to solve this modern-day environmental issue in an environmentally friendly way. The upcoming section will provide insight into phytoremediation and its efficiency under various conditions.

## 2. Phytoremediation

Phytoremediation is an effective method to counter the bottlenecks of other remediation strategies including inefficiency, cost, and secondary pollutant generation. It helps in absorbing these ionic pollutants from soil and water [34]. This technology is not only economic but also aesthetic [16]. Studies conducted to enhance the rate and efficiency of phytoremediation techniques over the last few decades help us to compare various strategies and decide the most efficient way to tackle the heavy metal problem.

**Figure 1:**
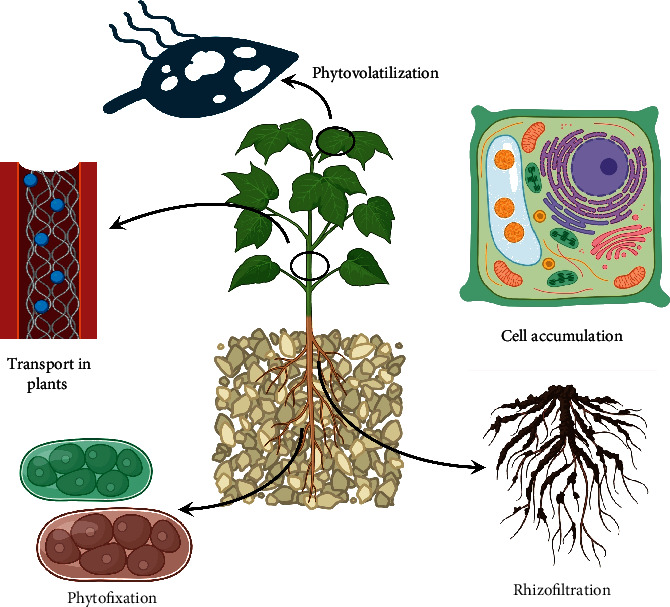
The diagram shows different phytoremediation strategies.

Phytoremediation exploits the root and shoot system of plants to interact with different contaminants. Plants themselves have significant tolerance for pollutants and heavy metals, while in some cases, heavy metals may have a toxic effect on growth and physiology. Recent studies have shown that *Lemna valdiviana* and *Ulva ohnoi* can remediate substantial concentrations of As (pH 6.3–7.0) and Cd, respectively, without any effects on the growth of the plant [33]. Some of the stimulators, amendments, and optimization approaches might help in improving the tolerance and survival rate of plants. Certain natural zeolites such as clinoptilolite and mordenite have helped in reducing As concentrations in shoots by 87% to prevent the entry of As into the food chain, thus making the remediation process not only environmentally friendly but also less wasteful, as it prevents the destruction of the crop after harvest [35]. The phytoremediation approach exploited multiple mechanisms including phytostabilization, phytoextraction, phytovolatization, and phytofiltration for heavy metal removal (Figure 1). The mechanisms are discussed below in detail.

## 3. Phytostabilization

**Figure 2:**
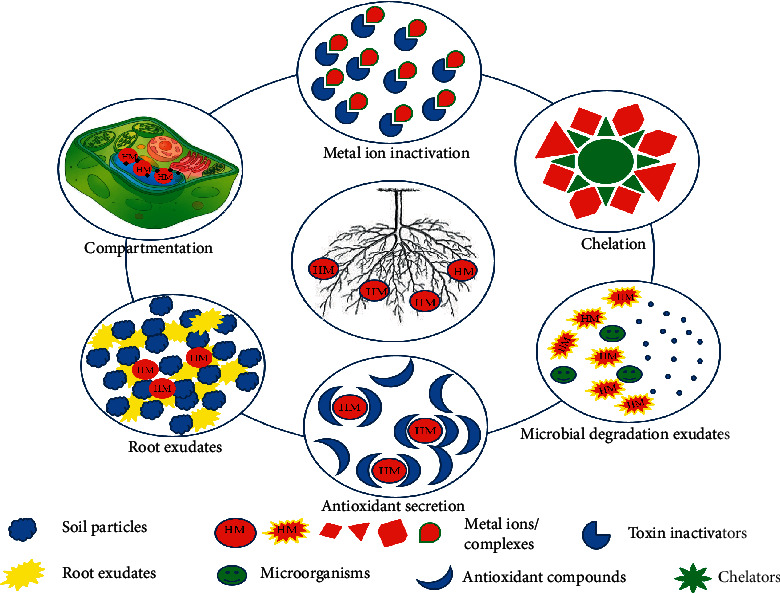
Phytostabilization of metal ions and complexes.

Phytostabilization deals with heavy metal pollutants by forming residue-bound molecules/complexes inside the root. In other words, the contaminants are immobilized within the roots or in the rhizosphere through adsorption, precipitation, and complexation [33, 36]. Heavy metal-tolerant species of plants including *Arabidopsis arenosa*, *A. halleri*, *Deschampsia cespitosa*, *Silene vulgaris* [37], *Zea mays, Brassica napus* [38] cutback the bioavailability of the contaminants in soil and minimize its leaching into groundwater sources, thus thwarting the entrance of the toxicants into the food chain [34]. Phytostabilization may be considered a slow process and is appropriate for low-value areas where nonagronomic plant species need to be used. These areas may include green spaces and ecosystem services [39]. Phytostabilization may be augmented by the addition of soil amendments such as manures, biosolids, and also bio-inoculants (bacteria and fungi) [40]. The evaluation of these strategies can be done through the change in soil health, pH, texture, organic matter content, and redox potential. Ecotoxicity tests such as root elongation, phytotoxicity assays, microbial activity, and enzymatic activity are also important evaluations that need to be considered for an all-round soil health profile [39]. In soil, rhizobacteria is a group of microorganisms that reside in close proximity to the root system and, hence, can play a crucial role in phytostabilizing the heavy metals. Gao et al. [41] conducted a study on phytostabilization of heavy metals by *Sasa argenteostriata* (Regel) E.G. Camu using two sets of Pb, Zn, and Cd (low and high concentrations). The analysis suggested that biological concentration factor (BCF) of *S. argenteostriata* raised with concentration of pollutants, while translocation factor reduced. Among all three heavy metals, Pb has shown the highest mobility in rhizosphere, and the residual concentration of Zn and Cd was high in rhizosphere due to low translocation. It was also found that rhizospheric bacteria have a crucial role in phytostabilization and *S. argenteostriata*-modulated rhizobacterial community by regulating the pH of rhizospheric soil. Wang et al. [42] also reported that the addition of soil amendments including spent mushroom compost and attapulgite improved the pH, fertility, and water retention of the manganese slag. The planting of *Koelreuteria paniculata* further aids in reducing the bioavailability of heavy metals and lowers the movement of Mn, Pb, and Zn via runoff by 15.7%, 8.4%, and 10.2%, respectively. The plant has changed the microbial population to improve the rhizosphere stability by augmenting beneficial fungi and suppressed the pathogenic fungi. The phytostabilization of pollutants works through six different mechanisms as mentioned in Figure 2.

Secretion of exudates such as saccharides, organic acids, proteins, peptides, and amino acids via roots enables the accumulation and stabilization of metal pollutants [38, 43], whereas metalloproteins such as phytochelatins and metallothioneins assist in chelating metal ions from the surrounding polluted soil [38, 44]. Microbial degradation of pollutants in root proximity also assists in immobilizing the heavy metal pollutants around the rhizosphere [38, 45]. Homogalacturonan (a pectin-binding that binds and deactivates metal ions in the cell wall) performs cell wall protective functions [34, 38]. Antioxidants such as superoxides, ascorbic acid, glutathione, and catalase help in countering the oxidative stress of metal ions and pollutants [34, 38, 46, 47]. The compartmentation of pollutants in vacuoles is another mechanism that helps in the immobilization of heavy metal pollutants [34, 38]. All these mechanisms are cumulatively responsible for effective and efficient heavy metal removal. Based on the activity of the abovementioned mechanisms, different plants have different tolerance ranges. *Imperata cylindrica* is seen to have a high tolerance for Cu at 300 mg.kg^−1^ [46]. The potential of metal ion chelation is also observed in maize against Cd in soil, by the addition of metal chelators such as ethylenediamine tetra-acetic acid and diethylenetriacetic acid [48].

## 4. Phytofiltration

**Figure 3:**
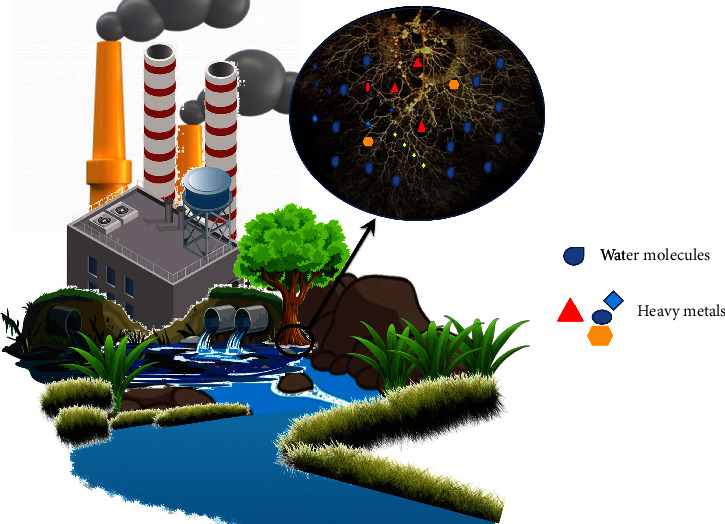
Trapping of pollutants by plant roots (rhizofiltration).

Phytofiltration is the filtration of heavy metals and pollutants from wastewater using shoots (caulofiltration), roots (rhizofiltration), and seedlings (blastofiltration) [34]. In rhizofiltration, the roots act as a filter, trapping, and accumulating heavy metal pollutants (Figure 3) [49]. In addition to filtration, the root exudates can further change the pH of the rhizosphere, leading to the precipitation of the pollutants and immobilizing them [34]. Water hyacinths, cattails, and azolla are commonly employed for this purpose [49]. The aquatic plant *Typha angustifolia* also exhibits a great capacity for rhizofiltration. It is capable of absorbing 4,941.1–14,109.4 mg of Cd and 14,039.3–59,360.8 mg of Zn per plant [50]. Terrestrial plants such as *B. Juncea* and *H. Annus* are also popularly used for rhizofiltration due to their longer and hairy root system. After these plants are saturated with the pollutants, they are harvested and disposed of [34]. Singh et al. have identified *Canna*, *Typha*, and *Eichhornia* [51] as potential phytoremediators for removing more than 90% heavy metals including Ni, Cu, Cd, Cr, and Pb by rhizofiltration. Sitarska et al. [52] also reported the removal of Hg by *Salvinia natans* via rhizofiltration with around 94% removal rate. The study also confirmed the toxic effect of Hg on plants as total protein and chlorophyll concentrations were reduced by 30% and 54%, respectively.

## 5. Phytovolatilization

This phytoremediation strategy can be employed in the case of heavy metals such as Se, Hg, and As and also for the detoxification of organic pollutants. It uses plants that convert pollutants into volatile molecules and complexes and release them into the atmosphere through surfaces like leaves [34]. Phytovolatilization has two forms: direct and indirect. Direct phytovolatilization is where the plant uptakes the pollutants, translocating them to the leaves where the pollutants are transformed and released into the atmosphere through transpiration. Water is the biggest source of nutrients for plants as well as for pollutants and contaminants. Pollutants move along with water and are taken up by plants through roots and distributed to other plant parts via conducting tissues. The aerial part of plants is in direct contact with sunlight and hence has a higher possibility to release volatile pollutants along with water through transpiration, which is referred to as direct phytovolatilization. Among intake pollutants, some are hydrophobic and need to follow the other path as the pathway of transpiration and direct phytovolatilization are different, especially for hydrophobic contaminants that need to cross cutin or suberin (hydrophobic barriers of the epidermis and woody dermal tissues). Some of the pollutants can also be released from the roots due to high volatile compounds flux in the root. This process of losing pollutants and contaminants from the root is referred to as indirect phytovolatilization. In other words, indirect phytovolatilization is the result of an increased flux of volatile contaminants from the subsurface due to root activities. Some of the factors that are responsible for increased flux are chemical transport via hydraulic redistribution, increase in soil permeability, advection of water toward the surface, etc. [53]. Ryegrass can be used for the volatilization of trifluralin compounds that are known to be residue-bound. Other plants that are commonly used for this process are *B. juncea* and *B. oleracea* for Se and *Polypogonmon speliensis* for As [36]. Another example is the use of poplar trees in the phytovolatilization of trichloroethylene (TCE), a common groundwater contaminant. Poplar trees absorb TCE through their roots and release it as a less toxic vapor [54].

## 6. Phytoextraction

Phytoextraction or phytoaccumulation simply refers to the absorption of heavy metal contaminants by roots and translocation and deposition into cell walls, cell membranes, and vacuoles of the shoots [36, 55]. Mainly, the metal cations that are absorbed by the plant form a metal–phytochelatin complex (M–PC), inside the plant, and are then translocated and stored in the vacuole [36]. The hyperaccumulator plants with metal-enriched biomass are further harvested and disposed of [55]. Thus, the recovery of heavy metals from biomass for recycling may be an alternative for sustainable heavy metal decontamination-cum-recycling. Specialized salt-excreting structures in halophytic plant species, found within the Tamaricaceae, Frankeniaceae, Poaceae, and Chenopodiaceae families, can be exploited to remove heavy metals from their leaves [56].

Plants such as *Amaranthus viridus*, *Cannabis sativa*, *Oenothera rosea*, *Chenopodium album*, *Sonchus asper*, *Datura stramonium*, *Nasturtium officinale*, *Polygonum maculosa*, *Conyza canadensis*, and *Xanthium stramonium* have also been reported for the phytoextraction of heavy metals such as Fe, Zn, Cr, Ni, and Cd from municipal wastewater [57]. The Agios Filippos Mine in Northeast Greece has been inactive for decades, and research on the soil quality in recent years has shown that even a broad area around the mine suffers from heavy metal contamination, particularly Cu, Mn, Zn, Pb, Cd, and As. A systematic planting of the plant *Thlaspi caerulescens* was proposed in addition to microbial and chemical amendments. This approach suggested the speculated recovery time to be 52 years, and the decontamination rate is expected to be 0.5 kg/ha/year for Cd and 20 kg/ha/year for Zn in a total decontaminated area of 2.2 km^2^ [58]. *Ceratophyllum demersum* has exceptionally proven itself in Egypt by reducing the leaching of Cr and PB by phytoaccumulation, where 95% of Pb and 84% of Cr removal were observed. Phytoextraction of Ni and Pb was observed in Pakistan by using the plant *Helianthus annuus*. More than 50% of removal, 17 mg/kg in plant tissue was seen over 2 decades [59]. Efforts have been made to estimate the potential of technology for the removal of heavy metal pollutants from water as well as soil (Table 3).

## 7. Challenges in Phytoremediation

Phytoremediation is a promising strategy for the cost-effective decontamination of polluted soil and water with heavy metals. However, it relies on the natural metabolic behavior of plant cells as well as microorganisms residing in close proximity (rhizosphere) to the said environment. Thus, being an in situ process, its application becomes challenging where the contaminated area needs to be converted into a green area, especially in heavily populated cities with space constraints. Also, the scale-up process is highly uncertain in terms of efficacy due to complex plant–soil–microbe interactions, and operational expenditure (OPEX) [75]. The adsorption capacity of plants and the toxic effect of heavy metals are also among the biggest challenges to the survival of plants [76]. In the case of high mortality of plants, the cost of operation will rise. It is also evident that phytoremediation is a slow process and requires large-scale cultivation of vegetation for up to several generations to eliminate pollutants from the affected area. The development of high tolerance lines is required, which need not only funds but also social acceptance. Therefore, government regulations and management plans might also raise an issue in using this strategy as specifically tailored plans need to be made for it to work appropriately [77]. Phytoremediation mostly refers to the process of accumulating heavy metals and pollutants within the plant biomass. It has the potential to be passed across the food chain upon consumption [78]. Besides, burning and valorization of plant biomass also tend to release bulk amounts of pollutants in the environment again.

## 8. Future Prospects and Nanophytoremediation

Even though phytoremediation is a promising approach for removing heavy metal contaminants from the soil, there are many shortcomings, as mentioned above, that need to be addressed. The future prospects of phytoremediation lie in widespread applications that can remove huge amounts of pollutants at a higher rate in comparatively less time. Some of the future directions are summarized below that can improve its widespread applicability:

• Increasing the performance of these plants by utilizing certain aids can prove helpful in improving the overall quality of our environment. It has been observed that the presence of amendments might have a conducive impact on phytoremediation. Natural amendments produced by processing biomass such as sugar beet and rice straw as well as chemical amendments such as ethylenediaminetetraacetic acid (EDTA) and sodium dodecyl sulfate (SDS) can act as soil amendments that can aid in phytoextraction and plant growth. They also help in enhancing the microbial activity around the rhizosphere, which aids the plant in metal bioavailability [36]. However, the use of natural amendments must be preferred to reduce the cost as well as possible toxicity on natural microflora. Hence, subsequent research becomes the need of the hour to develop more efficient strategies to improve the heavy metal removal along with enhancing symbiotic interactions between the hyperaccumulators and the microflora of the soil.
• The presence of other organic pollutants and native microflora also influences the efficiency and rate of phytoremediation. The mushroom has shown widespread potential to valorize organic waste especially lignocellulosic biomass and litter in soil for their growth and biomass gain. In addition, the growing fungi and mushrooms provide nutrients and growth promoters to plants to improve their growth [79, 80]. On the other hand, pollutants like microplastics obstruct the molecular flow in soil [81] and have a direct effect on plant growth as well as phytoremediation.
• Genetic engineering of hyperaccumulators approach can be used to enhance the heavy metal uptake and tolerance. The identification and characterization of genes and mechanisms involved in filtering the pollutants, accumulation, and conversion become a critical aspect that becomes a significant factor while overexpressing genes and the development of genetically engineered plants. It may also improve the performance of phytoremediation, especially by cutting down the immense time required. i.e., years in the case of woody plants.
• The advancements in molecular techniques enable precise selection and editing of gene/gene clusters by using CRISPR-Cas9, marker-assisted selection (MAS), TALENs, ZFNs, gene stacking, trait pyramiding, genome editing, RNA interference (RNAi), etc., to improve the expression of required traits [82].
• Integration of nanotechnology might also help in improving plant growth, metal accumulation, and combating the toxic effect of plants [83, 84]. Certain nanoparticles have been reported to enhance hyperaccumulator plants' biomass along with the uptake of pollutants by augmenting antioxidant activities [85] like zerovalent nanoparticles (nZNPs) that can remove heavy metals by providing an energy change [36]. Besides, nZNPs, multiwalled carbon nanotubes, and metal, and alloy-based nanoparticles have also been reported for the degradation of toxic organic contaminants and metal complexes including atrazine, 2,4-dinitrotoluene, lindane, chlorpyrifos, PCBs, pyrene, and TCE, and pentachlorophenol [86]. Extensive research is needed to explore more such nanoparticles and carbon dots to enhance the phytoremediation process along with the reduction in time required.
• Nowadays, most industrial processes are switching toward green and cost-effective feed or raw materials [87, 88]. The accumulation and storage of heavy metals and pollutants in plant biomass become a challenge as biomass releases a bulk amount of pollutants in nature after plants' death and during the valorization process. The proper solution is required to retrieve the heavy metals and salts for their further upcycling and recycling.

Practically, scientific research alone cannot overcome these large gaps for environmental pollutants, and heavy metal accumulation due to limited landscape, resources, and funding opportunities. Government interventions and industrial investment become equally important to solve these bottlenecks. Without the assistance and cooperation of the authorities of the state, it would be impossible to detoxify these contaminated environments. In addition, responsible behavior is also required to minimize the use of polluting chemicals in the required amount only, when its application becomes necessary.

## 9. Conclusion

Phytoremediation is an eco-friendly and sustainable approach for pollutant removal and degradation from soil as well as water. It relies on natural metabolic and development activities that allow plants to uptake pollutants and heavy metals followed by transformation to volatile form or accumulate within cells as complex or poor or no-toxicity. However, in some cases, the plant's low tolerance power, serious toxic impact on the plant's growth, and slow rate of detoxification have raised concerns about the poor performance of phytoremediation. Recent research efforts have integrated nanotechnology and nanomaterials to improve overall performance by changing the form of pollutants or immobilizing them on nanomaterials. However, the approach needs feasibility analysis for long-term as well as short-term impacts on the environment. The assessment must also be conducted for end valorization analysis for plant biomass.

## Figures and Tables

**Table 1:** Detection methods and toxicity of different heavy metals.

Heavy metal/s	Source	Toxic effect	Detection method/s	Detoxification strategy	References
Cadmium	Geological deposits, wildfires, fish and seafood, battery manufacturing, mining and welding, phosphate fertilizers	Carcinogenicity, damage to pulmonary and gastrointestinal tract, nephrotoxicity	Atomic absorption/emission/fluorescence spectroscopy, inductively coupled plasma–mass spectrometry, electrochemical analysis, colorimetry	Chemical precipitation, adsorption, ion exchange, solvent extraction, membrane filtration, chelation therapy, bioremediation	[19–21]
Mercury	Seafood contaminated with methylmercury, dental amalgams, transportation of mercury ores, mining	Severe health issues affecting nervous system, tremors, memory loss, psychiatric disturbances, miscarriages, birth defects	Atomic absorption/emission/fluorescence spectroscopy, inductively coupled plasma–mass spectrometry, electrochemical analysis, colorimetry	Chemical precipitation, coagulation-flocculation, adsorption, UV-electrooxidation, electro-fenton system, photocatalysis	[20–22]
Lead	Lead-based paints, contaminated soil, certain drinking water systems, batteries, pesticides	Developmental issues in children, cognitive impairments, lead poisoning, effects on kidney	Atomic absorption/emission/fluorescence spectroscopy, inductively coupled plasma–mass spectrometry, electrochemical analysis, colorimetry	Soil amendments, chelation therapy	[20, 21]
Arsenic	Paints, metal smelters, fossil fuels, pesticides, textile industry	Skin, lung and bladder cancer, skin lesions, cardiovascular issues, bronchitis	Atomic fluorescence spectroscopy, inductively coupled plasma–mass spectrometry	Coagulation, precipitation, membrane filtration, anion exchange	[23]
Chromium	Electroplating, leather tanning, stainless steel production, dyes	Lung cancer, ulceration, skin irritation, alteration of genetic material, vomiting, respiration problems	Electrochemical analysis, ion chromatography, atomic absorption spectroscopy, gas chromatography, high performance liquid chromatography	Redox transformation, electrocoagulation, bioremediation	[24, 25]
Zinc	Certain dietary supplements, occupational exposure in industries such as metal processing, metal plating, galvanization, paints	Skin corrosion, nausea, vomiting, diarrhea, damage to nervous membrane	Electrochemical analysis	Precipitation, bioremediation	[26]
Copper	Naturally in Earth's crust, industrial processes, pesticides, electronic waste, plumbing, areas with copper pipes, pigments, electroplating	Gastrointestinal issues such as nausea, vomiting, liver and kidney damage, Wilson's disease, severe anemia, brain damage	Electrochemical analysis	Electrowinning, electroextraction	[27, 28]
Nickel	Stainless steel production, batteries, zinc-based casting, fertilizers, metal refining	Skin irritation, dermatitis, allergic reactions, lung and nasal cancers, effects on kidney and cardiovascular system, immunotoxic, neurological problems	Fluorescence method	Chemical precipitation, bioremediation, chelation therapy, agricultural/soil amendments, electrocoagulation	[24, 29]

**Table 2:** Advantages and disadvantages of various waste and heavy metal removal and treatment methods.

Remediation techniques		Mechanism	Advantages	Disadvantages
Domain	Type of treatment			
Physical techniques	Filtration	Separate mixture based on the size and solubility	• Easy operation	• Soluble compounds cannot be separated by conventional system
			• Low capital investment	• Preferred for liquid system
			• High efficiency	• Large-scale operation is costly
	Sedimentation	Settling of suspended particles based on gravity	• Simple and inexpensive	• High toxicity;
			• Effective	• Sediment removal and processing is costly
				• Chelating agent use and recycling is itself costly
				• Ineffective against trivalent metal ions including Fe^3+^, Al^3+^, and Cr^3+^
	Absorption	Immobilization of pollutants on resin surface and pores	• Simple and cost-effective	• Production of adsorbent is costly
			• High removal efficiency	• Again, discharge waste after regeneration of waste
			• Effective at a wide range of pH	

Chemical techniques	Electro-catalytic oxido-reduction	Oxidation and reduction under the influence of electric field and catalyst	• Rapid and effective	• Formation of sludge or abduct material
				• Cost intensive
	Electro-deposition	High-energy electrons reduced metal ions into elements	• Rapid and effective	• Electrode cost is high
			• Can work at large scale as well	• Electrode replacement or regeneration is costly
	Flocculation	Form complex with pollutants and remove suspended material by precipitation or in the form of clump	• Easy to remove suspended particles and pollutants from liquid	• Suitable for liquid only
				• Use of chemical as flocculating agent for removal
				• Form sludge-like waste
	Dialysis and electrodialysis	Removal of solvents based on concentration based on concentration gradients. Electric field further improve the movement	• High rate of removal for charged metal ions under electric field	• Energy intensive
				• Scale-up is a tedious task
				• Only transfer pollutants from one phase to other

Biological techniques	Microbial accumulation	Microbes degrade complex and accumulate metals in cytoplasm	• Low-cost operation	• Only accumulate heavy metals in cell mass
			• Onsite and offsite operation possible	• Biomass degradation again releases pollutants
	Enzymatic degradation	Enzyme catalyze the degradation or biotransformation	• Enzyme recyclability and tolerance is a critical factor	• Cost intensive
			• Specific in action	• Complex process with multiple affecting factors
	Phytoremediation	Plants segregate selectively and accumulate within biomass in different organs	• Prolong storage of pollutants	• Slow in action
			• Effective and selective in action	• Dead matter valorization again releases bulk amount of pollutants in the environment
			• High tolerance	

**Table 3:** Phytoremediation of heavy metals from various sites.

Heavy metal/s	Source	Plant involved	Mechanism	Major findings	References
Cadmium	Industrial discharge, contaminated water and soil	*Brassica juncea, Helianthus annuus*	Accumulate Cd in their tissues through various mechanisms including ion uptake and sequestration	Indian mustard and sunflower are strong accumulators of Cd and effectively remove it from contaminated soil.	[60, 61]
Mercury	Natural deposits, coal-fired power plants, industrial processes	*Eichhornia crassipes, Lemna* spp.	Absorb and accumulate mercury from water through their root and shoot systems	High potential for removing Hg from aquatic systems	[62, 63]
Lead	Old paint, contaminated soil, industrial emissions	*Brassica juncea, Helianthus annuus*	Can stabilize lead in the root system and reduce its mobility through root uptake and binding	Ability of these plants to accumulate and immobilize lead in contaminated soil	[64, 65]
Arsenic	Naturally occurring in some regions, industrial processes	Ferns, *Helianthus annuus*	Accumulate As particularly in the roots via mechanisms such as uptake and translocation	Ability of certain ferns and sunflowers to accumulate and tolerate high levels of arsenic	[66, 67]
Chromium	Tanneries, electroplating, industrial discharges	*Brassica juncea*, willow trees	Accumulate and reduce hexavalent chromium (Cr(VI)) to less toxic trivalent chromium (Cr(III))	Effectiveness of these plants in reducing chromium in contaminated soil	[68, 69]
Zinc	Industrial effluents, mining, agriculture	*Thlapsi caerulescens*, willow trees	Hyperaccumulation, store Zn in their aboveground biomass	Potential in removal of Zn from contaminated soil	[70, 71]
Copper	Agricultural runoff, industrial discharges, mining	*Eichhornia crassipes*, willow trees	Accumulate and store Cu in their tissues	Ability to remove Cu from contaminated water and soil	[71, 72]
Nickel	Mining, metal plating, industrial activities	*Alyssum* spp., willow trees	Hyperaccumulation, absorb and store Ni in their tissues, mainly in the leaves	Potential in phytoremediation of Ni from contaminated soil	[73, 74]

## Data Availability

Most of the data used in the study are included in the manuscript. Further information is available with the corresponding author.

## References

[1] Briffa J., Sinagra E., Blundell R. (2020). Heavy Metal Pollution in the Environment and Their Toxicological Effects on Humans. *Heliyon*.

[2] Mazarji M., Bayero M. T., Minkina T. (2023). Nanomaterials in Biochar: Review of Their Effectiveness in Remediating Heavy Metal-Contaminated Soils. *Science of The Total Environment*.

[3] Riar J. K., Bhanot R., Hundal S. S. (2021). Assessment of Heavy Metals in Samples of Soil, Water, Vegetables, and Vital Organs of Rat (*Bandicota bengalensis*) Collected From Adjoining Areas of Polluted Water Body. *Water, Air, & Soil Pollution*.

[4] Liu X., Bai Z., Shi H., Zhou W., Liu X. (2019). Heavy Metal Pollution of Soils From Coal Mines in China. *Natural Hazards*.

[5] Yang Le, Wang J., Yang Y. (2022). Phytoremediation of Heavy Metal Pollution: Hotspots and Future Prospects. *Ecotoxicology and Environmental Safety*.

[6] Petrosyan V., Pirumyan G., Perikhanyan Y. (2019). Determination of Heavy Metal Background Concentration in Bottom Sediment and Risk Assessment of Sediment Pollution by Heavy Metals in the Hrazdan River (Armenia). *Applied Water Science*.

[7] Xiang M., Yan Li, Yang J. (2021). Heavy Metal Contamination Risk Assessment and Correlation Analysis of Heavy Metal Contents in Soil and Crops. *Environmental Pollution*.

[8] Mahdi B.-M., Naseri K., Tahergorabi Z., Khazdair M. R., Sadeghi M. (2021). Toxic Mechanisms of Five Heavy Metals: Mercury, Lead, Chromium, Cadmium, and Arsenic. *Frontiers in Pharmacology*.

[9] Goyal V. C., Singh O., Singh R., Chhoden K., Malyan S. K. (2022). Appraisal of Heavy Metal Pollution in the Water Resources of Western Uttar Pradesh, India and Associated Risks. *Environmental Advances*.

[10] Hoque M. M., Islam A., Islam A. R. M. T., Pal S. C., Mahammad S., Alam E. (2023). Assessment of Soil Heavy Metal Pollution and Associated Ecological Risk of Agriculture Dominated Mid-Channel Bars in a Subtropical River Basin. *Scientific Reports*.

[11] Iqbal H. H., Siddique A., Qadir A., Rashid Ahmad S., Liess M., Shahid N. (2024). Human Health and Ecology at Risk: A Case Study of Metal Pollution in Lahore, Pakistan. *Environmental Sciences Europe*.

[12] Aslam A., Naz A., Shah S. S. H. (2023). Heavy Metals Contamination in Vegetables Irrigated With Wastewater: A Case Study of Underdeveloping Regions of Pakistan. *Environmental Geochemistry and Health*.

[13] Huang J.-Li, Li Z.-Y., Mao J.-Y. (2024). Contamination and Health Risks Brought by Arsenic, Lead and Cadmium in a Water-Soil-Plant System Nearby a Non-Ferrous Metal Mining Area. *Ecotoxicology and Environmental Safety*.

[14] Fernández-Martínez R., Corrochano N., Álvarez-Quintana J., Ordóñez A., Álvarez R., Rucandio I. (2024). Assessment of the Ecological Risk and Mobility of Arsenic and Heavy Metals in Soils and Mine Tailings From the Carmina Mine Site (Asturias, NW Spain). *Environmental Geochemistry and Health*.

[15] Paniagua-López M., García-Robles H., Aguilar-Garrido A., Romero-Freire A., Lorite J., Sierra-Aragón M. (2024). Vegetation Establishment in Soils Polluted by Heavy Metal(Loid)s After Assisted Natural Remediation. *Plant and Soil*.

[16] Liu L., Li W., Song W., Guo M. (2018). Remediation Techniques for Heavy Metal-Contaminated Soils: Principles and Applicability. *Science of the Total Environment*.

[17] WHO (2023). *Lead Poisoning*.

[18] Aradhi K. K., Mallesh Dasari B., Banothu D., Manavalan S. (2023). Spatial Distribution, Sources and Health Risk Assessment of Heavy Metals in Topsoil Around Oil and Natural Gas Drilling Sites, Andhra Pradesh, India. *Scientific Reports 2023*.

[19] Khan Z., Elahi A., Bukhari D. A., Rehman A. (2022). Cadmium Sources, Toxicity, Resistance and Removal by Microorganisms-A Potential Strategy for Cadmium Eradication. *Journal of Saudi Chemical Society*.

[20] Liu X., Yu K., Zhang H. (2020). A Portable Electromagnetic Heating-Microplasma Atomic Emission Spectrometry for Direct Determination of Heavy Metals in Soil. *Talanta*.

[21] Perelonia K. B. S., Benitez K. C. D., Banicod R. J. S., Tadifa G. C., Cambia F. D., Montojo U. M. (2021). Validation of an Analytical Method for the Determination of Cadmium, Lead and Mercury in Fish and Fishery Resources by Graphite Furnace and Cold Vapor Atomic Absorption Spectrometry. *Food Control*.

[22] Pavithra, Grace K., SundarRajan P., Senthil Kumar P., Rangasamy G. (2023). Mercury Sources, Contaminations, Mercury Cycle, Detection and Treatment Techniques: A Review. *Chemosphere*.

[23] Rathi B. S., Senthil Kumar P. (2021). A Review on Sources, Identification and Treatment Strategies for the Removal of Toxic Arsenic From Water System. *Journal of Hazardous Materials*.

[24] Akbal F., Camcı S. (2011). Copper, Chromium and Nickel Removal From Metal Plating Wastewater by Electrocoagulation. *Desalination*.

[25] Li M.-hao, Gao X.-yan, Li C. (2020). Isolation and Identification of Chromium Reducing Bacillus Cereus Species From Chromium-Contaminated Soil for the Biological Detoxification of Chromium. *International Journal of Environmental Research and Public Health*.

[26] Essa A. M. M., Mohamed A. Al A., Khatib S. I. (2018). Metal Transformation as a Strategy for Bacterial Detoxification of Heavy Metals. *Journal of Basic Microbiology*.

[27] Fathima A., Tang J. Y. B., Giannis A., Ilankoon I. M. S. K., Chong M. N. (2022). Catalysing Electrowinning of Copper from E-Waste: A Critical Review. *Chemosphere*.

[28] Tang J., Su M., Wu Q. (2019). Highly Efficient Recovery and Clean-Up of Four Heavy Metals from MSWI Fly Ash by Integrating Leaching, Selective Extraction and Adsorption. *Journal of Cleaner Production*.

[29] Mustafa A., Zulfiqar U., Mumtaz M. Z. (2023). Nickel (Ni) Phytotoxicity and Detoxification Mechanisms: A Review. *Chemosphere*.

[30] Kumar M., Seth A., Singh A. K., Singh Rajput M., Sikandar M. (2021). Remediation Strategies for Heavy Metals Contaminated Ecosystem: A Review. *Environmental and Sustainability Indicators*.

[31] Rahman Z., Singh V. P. (2020). Bioremediation of Toxic Heavy Metals (THMs) Contaminated Sites: Concepts, Applications and Challenges. *Environmental Science and Pollution Research*.

[32] Ali H., Khan E., Sajad M. A. (2013). Phytoremediation of Heavy Metals—Concepts and Applications. *Chemosphere*.

[33] Tang K. Ho D. (2023). Phytoremediation: Where Do We Go from Here?. *Biocatalysis and Agricultural Biotechnology*.

[34] Yan An, Wang Y., Tan S. N., Yusof M. L. M., Ghosh S., Zhong C. (2020). Phytoremediation: A Promising Approach for Revegetation of Heavy Metal-Polluted Land. *Frontiers in Plant Science*.

[35] Budianta W. (2021). The Use of Natural Zeolites From Gunungkidul, Indonesia for Preventing Arsenic Pollution of Soils and Plants. *IOP Conference Series: Earth and Environmental Science*.

[36] Kafle A., Timilsina A., Gautam A., Adhikari K., Bhattarai A., Aryal N. (2022). Phytoremediation: Mechanisms, Plant Selection and Enhancement by Natural and Synthetic Agents. *Environmental Advances*.

[37] Borymski S. ., Cycoń M., Beckmann M., Mur L. A. J., Piotrowska-Seget Z. (2018). Plant Species and Heavy Metals Affect Biodiversity of Microbial Communities Associated With Metal-Tolerant Plants in Metalliferous Soils. *Frontiers in Microbiology*.

[38] Bakshe P., Jugade R. (2023). Phytostabilization and Rhizofiltration of Toxic Heavy Metals by Heavy Metal Accumulator Plants for Sustainable Management of Contaminated Industrial Sites: A Comprehensive Review. *Journal of Hazardous Materials Advances*.

[39] Lacalle R. G., Bernal M. P., Álvarez-Robles M. J., Clemente R. (2023). Phytostabilization of Soils Contaminated With as, Cd, Cu, Pb and Zn: Physicochemical, Toxicological and Biological Evaluations. *Soil & Environmental Health*.

[40] Bolan N. S., Park J. H., Robinson B., Naidu R., Huh K. Y., Donald L., Sparks (2011). Chapter Four-Phytostabilization: A Green Approach to Contaminant Containment. *Advances in Agronomy*.

[41] Gao Y., Jiang M., Luo Z. (2024). *Sasa Argenteostriata*–A Potential Plant for Phytostabilization Remediation of Lead-Zinc Tailing-Contaminated Soil. *Ecotoxicology and Environmental Safety*.

[42] Wang J., Delavar M. A. (2024). Modelling Phytoremediation: Concepts, Methods, Challenges and Perspectives. *Soil & Environmental Health*.

[43] Sardans J., Lambers H., Preece C., Alrefaei A. F., Penuelas J. (2023). Role of Mycorrhizas and Root Exudates in Plant Uptake of Soil Nutrients (Calcium, Iron, Magnesium, and Potassium): Has the Puzzle Been Completely Solved?. *The Plant Journal*.

[44] Anderson A. J., Hortin J. M., Jacobson A. R., Britt D. W., McLean J. E. (2023). Changes in Metal-Chelating Metabolites Induced by Drought and a Root Microbiome in Wheat. *Plants*.

[45] Abo-Alkasem M. I., Hassan Ne’mat H., Elsoud M. M. A. (2023). Microbial Bioremediation as a Tool for the Removal of Heavy Metals. *Bulletin of the National Research Centre*.

[46] Vidal C., Ruiz A., Ortiz J. (2020). Antioxidant Responses of Phenolic Compounds and Immobilization of Copper in Imperata Cylindrica, a Plant With Potential Use for Bioremediation of Cu Contaminated Environments. *Plants*.

[47] Ahmad N., Naeem M., Ali H. (2023). From Challenges to Solutions: The Impact of Melatonin on Abiotic Stress Synergies in Horticultural Plants via Redox Regulation and Epigenetic Signaling. *Scientia Horticulturae*.

[48] Yang Q., Yang C., Yu H., Zhao Z., Bai Z. (2021). The Addition of Degradable Chelating Agents Enhances Maize Phytoremediation Efficiency in Cd-Contaminated Soils. *Chemosphere*.

[49] Carvajal L., Benavides L., Rodriguez R., Serrezuela W. R. (2018). Extraction in Laboratory of Heavy Metals Through Rhizofiltration Using the Plant Zea Mays (Maize). *International Journal of Applied Environmental Sciences*.

[50] Woraharn S., Meeinkuirt W., Phusantisampan T., Chayapan P. (2021). Rhizofiltration of Cadmium and Zinc in Hydroponic Systems. *Water, Air, & Soil Pollution*.

[51] Singh S., Kaushik A., Bendi A., Chetal A., Ramakrishna D. S., Lakshmi Praveen P. (2024). Constructed Wetlands as Bioeconomic Solutions: Rhizofiltration With Macrophytes for Heavy Metal Removal. *Emergent Materials*.

[52] Sitarska M., Traczewska T., Hołtra A., Zamorska-Wojdyła D., Filarowska W., Hanus-Lorenz B. (2023). Removal of Mercury From Water by Phytoremediation Process With *Salvinia natans*(L.) All. *Environmental Science and Pollution Research International*.

[53] Limmer M., Burken J. (2016). Phytovolatilization of Organic Contaminants. *Environmental Science and Technology*.

[54] Doty S. L., Freeman J. L., Cohu C. M. (2017). Enhanced Degradation of TCE on a Superfund Site Using Endophyte-Assisted Poplar Tree Phytoremediation. *Environmental Science & Technology*.

[55] Yanitch A., Kadri H., Frenette-Dussault C. ., Joly S., Pitre F. E., Labrecque M. (2020). A Four-Year Phytoremediation Trial to Decontaminate Soil Polluted by Wood Preservatives: Phytoextraction of Arsenic, Chromium, Copper, Dioxins and Furans. *International Journal of Phytoremediation*.

[56] Naikoo M. I., Kafeel U., Naushin F., Ahmad Khan F., Grigore M.-N. (2020). Halophytes in India and Their Role in Phytoremediation. *Handbook of Halophytes: From Molecules to Ecosystems towards Biosaline Agriculture*.

[57] Irshad M., Ruqia B., Hussain Z. (2015). Phytoaccumulation of Heavy Metals in Natural Vegetation at the Municipal Wastewater Site in Abbottabad, Pakistan. *International Journal of Phytoremediation*.

[58] Giakoumatos S. D. V., Kopsidas O. N. (2021). Soil Phytoremediation—A Case Study in Greece. *Journal of Environmental Science and Engineering*.

[59] Cristián Raziel D.-G., Madariaga-Navarrete A., Fernández-Cortés J. M. (2021). Advances and Applications of Water Phytoremediation: A Potential Biotechnological Approach for the Treatment of Heavy Metals From Contaminated Water. *International Journal of Environmental Research and Public Health*.

[60] Gurajala H. K., Cao X., Lin T., Ramesh T. M., Lu M., Yang X. (2019). Comparative Assessment of Indian Mustard (*Brassica Juncea* L.) Genotypes for Phytoremediation of Cd and Pb Contaminated Soils. *Environmental Pollution*.

[61] Mathur J., Chauhan P., Srivastava S. (2023). Comparative Evaluation of Cadmium Phytoremediation Potential of Five Varieties of *Helianthus Annuus* L. *International Journal of Phytoremediation*.

[62] Li S.-X., Feng-Ying Z., Yang H., Ni J.-C. (2011). Thorough Removal of Inorganic and Organic Mercury From Aqueous Solutions by Adsorption on Lemna Minor Powder. *Journal of Hazardous Materials*.

[63] Monroy-Licht A., Méndez-Cuadro D., Olivero-Verbel J. (2022). Elemental Mercury Accumulation in Eichhornia Crassipes (Mart.) Solms-Laubach. *Environmental Science and Pollution Research*.

[64] Al-Jobori K., Kadhim A. (2019). Evaluation of Sunflower (Helianthus Annuus L.) for Phytoremediation of Lead Contaminated Soil. *Journal of Pharmaceutical Sciences and Research*.

[65] Bassegio C., Campagnolo M. A., Schwantes D. (2020). Growth and Accumulation of Pb by Roots and Shoots of Brassica Juncea L. *International Journal of Phytoremediation*.

[66] Cantamessa S., Massa N., Gamalero E., Berta G. (2020). Phytoremediation of a Highly Arsenic Polluted Site, Using Pteris Vittata L. and Arbuscular Mycorrhizal Fungi. *Plants*.

[67] Sharma M., Mathur J., Goswami P. (2023). Ultrastructural Analysis and Biochemical Evaluation Provide Evidence for Arsenic Reclamation From Contaminated Soil Through Helianthus Annuus L. *Acta Physiologiae Plantarum*.

[68] Dotaniya M. L., Rajendiran S., Saurabh K., Saha J. K., Dotaniya C. K., Patra A. K. (2023). Immobilization of Chromium Bioavailability Through Application of Organic Waste to Indian Mustard (Brassica Juncea) under Chromium-Contaminated Indian Soils. *Environmental Monitoring and Assessment*.

[69] Quaggiotti S., Barcaccia G., Schiavon M. (2007). Phytoremediation of Chromium Using Salix Species: Cloning ESTs and Candidate Genes Involved in the Cr Response. *Gene*.

[70] Frey B., Keller C., Zierold K. (2000). Distribution of Zn in Functionally Different Leaf Epidermal Cells of the Hyperaccumulator *Thlaspi Caerulescens*. *Plant, Cell and Environment*.

[71] Labrecque M., Hu Y., Vincent G., Shang K. (2020). The Use of Willow Microcuttings for Phytoremediation in a Copper, Zinc and Lead Contaminated Field Trial in Shanghai, China. *International Journal of Phytoremediation*.

[72] Mahfooz Y., Yasar A., Islam Q. Ul, Rasheed R., Naeem U., Mukhtar S. (2021). Field Testing Phytoremediation of Organic and Inorganic Pollutants of Sewage Drain by Bacteria Assisted Water Hyacinth. *International Journal of Phytoremediation*.

[73] Adamidis G. C., Aloupi M., Kazakou E., Dimitrakopoulos P. G. (2014). Intra-Specific Variation in Ni Tolerance, Accumulation and Translocation Patterns in the Ni-Hyperaccumulator Alyssum Lesbiacum. *Chemosphere*.

[74] Korzeniowska J., Stanislawska-Glubiak E. (2019). Phytoremediation Potential of Phalaris Arundinacea, Salix Viminalis and Zea Mays for Nickel-Contaminated Soils. *International journal of Environmental Science and Technology*.

[75] Wang H., Liu H., Su R., Chen Y. (2024). Phytostabilization of Heavy Metals and Fungal Community Response in Manganese Slag Under the Mediation of Soil Amendments and Plants. *Toxics*.

[76] Sodhi, Kaur K., Mishra L. C., Singh C. K., Kumar M. (2022). Perspective on the Heavy Metal Pollution and Recent Remediation Strategies. *Current Research in Microbial Sciences*.

[77] Lee S. H., Park H., Kim J. G. (2023). Current Status of and Challenges for Phytoremediation as a Sustainable Environmental Management Plan for Abandoned Mine Areas in Korea. *Sustainability*.

[78] Amanullah M., Wang P., Ali A. (2016). Challenges and Opportunities in the Phytoremediation of Heavy Metals Contaminated Soils: A Review. *Ecotoxicology and Environmental Safety*.

[79] Khan A. A., Lu Li-X., Yao F.-J. (2023). Characterization, Antioxidant Activity, and Mineral Profiling of Auricularia Cornea Mushroom Strains. *Frontiers in Nutrition*.

[80] Khan A. A., Muhammad M. J., Muhammad I. (2019). Modulation of Agronomic and Nutritional Response of Pleurotus Eryngii Strains by Utilizing Glycine Betaine Enriched Cotton Waste. *Journal of the Science of Food and Agriculture*.

[81] Khan A. A., Iqbal B., Jalal A. (2024). Advanced Molecular Approaches for Improving Crop Yield and Quality: A Review. *Journal of Plant Growth Regulation*.

[82] Khan I., Tariq M., Alabbosh K. F. (2024). Soil Microplastics: Impacts on Greenhouse Gasses Emissions, Carbon Cycling, Microbial Diversity, and Soil Characteristics. *Applied Soil Ecology*.

[83] Singh, Brajesh K. (2009). Organophosphorus-Degrading Bacteria: Ecology and Industrial Applications. *Nature Reviews Microbiology*.

[84] El-Saadony, Mohamed T., Saad M. A. (2022). Role of Nanoparticles in Enhancing Crop Tolerance to Abiotic Stress: A Comprehensive Review. *Frontiers in Plant Science*.

[85] Ojuederie O. B., Amoo A. E., Owonubi S. J., Ayangbenro A. S., Pandey V. (2022). Chapter 6-Nanoparticles-Assisted Phytoremediation: Advances and Applications. *Assisted Phytoremediation*.

[86] Gul M. Z., Rupula K., Beedu S. R., Bhat R. A., Policarpo Tonelli F. M., Hamid Dar G., Hakeem K. (2022). Chapter 6-Nano-Phytoremediation for Soil Contamination: An Emerging Approach for Revitalizing the Tarnished Resource. *Phytoremediation*.

[87] Ahuja V., Bhatt A. K., Mehta S., Sharma V., Kumari Rathour R., Sheetal (2022). Xylitol Production by Pseudomonas Gessardii VXlt-16 From Sugarcane Bagasse Hydrolysate and Cost Analysis. *Bioprocess and Biosystems Engineering*.

[88] Dasgupta D., Bandhu S.eetal, Adhikari D. K., Ghosh D. (2017). Challenges and Prospects of Xylitol Production With Whole Cell Bio-Catalysis: A Review. *Microbiological Research*.

